# Supplementary material for: The Rho-guanine nucleotide exchange factor PDZ-RhoGEF governs susceptibility to diet-induced obesity and type 2 diabetes
Source: eLife. 2015 Oct 29;4:e06011. doi: 10.7554/eLife.06011 (PMC4709268; doi:10.7554/eLife.06011)
Supplement: Supplementary file 1. — DOI: http://dx.doi.org/10.7554/eLife.06011.028 [file elife-06011-supp1.docx]

**Supplementary File 1. Composition of high-fat diet (Harlan Tekld TD-01435)**

| Formula | **g/Kg** | **Representative Ingredients** |
| --- | --- | --- |
| Casein | 240.0 | Ground corn |
| DL-Methionine | 3.6 | Dehulled soybean meal |
| Maltodextrin | 150.0 |  |
| Corn Starch | 123.9 | Ground oats |
| Sucrose | 130.0 | Wheat middlings |
| Soybean Oil | 142.5 |  |
| Coconut Oil, hydrogenated | 80.0 |  |
| Cellulose | 61.153 | Corn gluten meal |
| Mineral Mix, AIN-93G-MX (TD 94046) | 42 |  |
| Calcium Phosphate, dibasic CaHPO_4_ | 4.2 |  |
| Vitamin Mix, AIN-93-VX (TD 94047) | 16.8 | Iodized salt |
| Choline Bitartrate | 5.8 |  |
| Vitamin K, phylloquinone | 0.002 |  |
| TBHQ (antioxidant) | 0.045 |  |
|  |  |  |
| Nutrient Information | **High Fat Diet (2025 kcal/kg)** |  |
|  | **% of Kcal from** |  |
| Protein | 19.0 |  |
| Carbohydrate | 36.0 |  |
| Fat | 45.0 |  |
